# SAMHD1: a new insight into HIV-1 restriction in myeloid cells

**DOI:** 10.1186/1742-4690-8-55

**Published:** 2011-07-08

**Authors:** Corine St Gelais, Li Wu

**Affiliations:** 1 Center for Retrovirus Research, Department of Veterinary Bioscience, The Ohio State University, 1900 Coffey Road, Columbus, OH 43210, USA

## Abstract

Human myeloid-lineage cells are refractory to HIV-1 infection. The Vpx proteins from HIV-2 and sooty mangabey SIV render these cells permissive to HIV-1 infection through proteasomal degradation of a putative restriction factor. Two recent studies discovered the cellular protein SAMHD1 to be this restriction factor, demonstrating that Vpx induces proteasomal degradation of SAMHD1 and enhances HIV-1 infection in myeloid-lineage cells. SAMHD1 functions as a myeloid-cell-specific HIV-1 restriction factor by inhibiting viral DNA synthesis. Here we discuss the implications of these findings in delineating the mechanisms of HIV-1 restriction in myeloid-lineage cells and the potential role of Vpx in lentiviral pathogenesis.

## Introduction

Myeloid-lineage cells, including monocytes, dendritic cells (DCs) and macrophages, play a multifaceted role in HIV-1 initial infection and viral dissemination; however, these cell types are restrictive to post-entry HIV-1 infection *in vitro *[1,2]. For gene therapy purposes, transduction of human DCs with an HIV-1-derived lentiviral vector can be significantly enhanced by preincubation with virus-like particles derived from SIV [3]. Subsequent studies indicated that Vpx proteins from sooty mangabey SIV (SIVsm) and HIV-2 lineages efficiently enhance HIV-1 infection in human DCs and promote the accumulation of full-length viral DNA [4]. Further studies from several laboratories suggested that Vpx, similar to HIV-1 Vpr, interacts with the DCAF1 component of the CUL4A/DDB1 and E3 ubiquitin ligase complex (reviewed in [5,6]). However, only SIVsm/HIV-2 Vpx can efficiently enhance HIV-1 infection in DCs and macrophages [5]. These studies led to the hypothesis that Vpx targets a putative HIV-1 restriction factor for proteasomal degradation in myeloid cells through the E3 ubiquitin ligase complex [5,6], prompting the search for the unknown HIV-1 restriction factor in human myeloid cells that is counteracted by Vpx.

SIVsm, SIVsm-derived rhesus macaque SIV (SIVmac), and HIV-2 encode both Vpr, a homologue of the HIV-1 Vpr protein, and Vpx, a protein unique to the SIVsm lineage. *vpx *has likely evolved via duplication of the primate lentivirus *vpr *gene [5]. Early studies have demonstrated that macaques infected with Vpx-defective SIVmac or SIVsm had decreased viremia, impaired viral replication, and slower AIDS progression compared to wild-type SIV-infected animals, thus revealing the importance of Vpx in SIV pathogenesis [7,8]. The important role of Vpx in lentiviral infection in myeloid-lineage cells *in vitro *and *in vivo *indicates that Vpx is not merely a functional copy of Vpr, but may possess a unique function. Although Vpx has been reported to facilitate nuclear import of viral DNA [5], the precise function of Vpx in lentiviral pathogenesis remains to be defined.

## New findings and discussion

Using mass spectrometry, Laguette *et al. *identified SAMHD1 as a novel Vpx-interacting protein purified from differentiated human monocytic THP-1 cells that express tagged Vpx [9]. The rationale for using THP-1 cells was based on the previous work that differentiated THP-1 cells can be rendered more permissive to HIV-1 infection by transduction of SIVsm/HIV-2 Vpx-containing virus-like particles derived from SIVmac [5]. SAMHD1 is expressed in non-permissive cells, including THP-1 cells, primary monocytes, monocyte-derived macrophages and DCs, while permissive CD4^+ ^T cells and monocytic U937 cells do not express endogenous SAMHD1 [9], suggesting an inverse correlation between SAMHD1 expression and permissiveness to HIV-1 infection. Moreover, silencing of SAMHD1 in non-permissive cells (THP-1 cells and DCs) alleviates HIV-1 restriction, and over-expression of SAMHD1 in permissive cells (HeLa cells and U937 cells) inhibits HIV-1 infection [9].

By contrast, Hrecka and colleagues identified SAMHD1 from HEK 293T cells expressing tagged Vpx in a proteomic screen using multidimensional protein identification technology [10]. They demonstrated that Vpx relieves the inhibition of HIV-1 infection in monocyte-derived macrophages by mediating proteasome-dependent degradation of SAMHD1 through the CUL4A/DCAF1 E3 ubiquitin ligase [10]. Both studies confirmed that Vpx interacts with SAMHD1 and induces proteasomal degradation of SAMHD1 in THP-1 cells or macrophages, which can be restored by treatment with a proteasome inhibitor [9,10].

**Figure 1:**
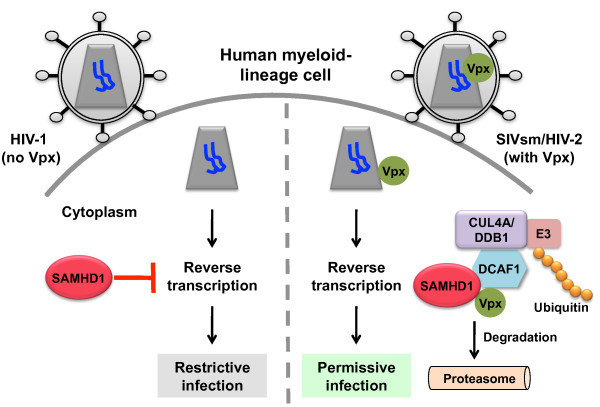
**Vpx interacts with the E3 ubiquitin ligase complex to target the restriction factor SAMHD1 for proteasomal degradation**. Human myeloid-lineage cells that are non-permissive to HIV-1 infection express high levels of SAMHD1, which appears to act early in infection at the reverse transcription step. HIV-1 has not evolved a viral antagonist to counter this restriction; however, SIVsm/SIVmac and HIV-2 express Vpx to circumvent this restriction. Vpx targets SAMHD1 using the host cell E3 ubiquitin ligase complex, in which Vpx interacts with the DCAF1 subunit of the CUL4A/DDB1 ubiquitin ligase to degrade SAMHD1 via the proteasome. This allows HIV-1 reverse transcription to occur and viral replication to complete.

The HD domains have putative nucelotidase and phosphodiesterase activities, and the highly conserved histidine (H) and aspartic acid (D) residues are critical for catalytic activity [11]. Indeed, Laguette *et al. *showed that over-expression of a HD domain mutant SAMHD1 in U937 cells fails to restrict HIV-1, suggesting that the phosphodiesterase activity of the HD domain is important for the restriction function of SAMHD1. Further analysis revealed that SAMHD1 blocks HIV-1 reverse transcription, as silencing SAMHD1 in THP-1 cells [9] and macrophages [10] increases the levels of viral DNA. Together, these studies suggested that SAMHD1 is the myeloid-cell specific HIV-1 restriction factor counteracted by Vpx [9,10] (Figure 1).

The new findings by Laguette *et al. *and Hrecka *et al. *have opened the door towards understanding the potential role of SAMHD1 in lentiviral pathogenesis. SAMHD1-mediated HIV-1 restriction in myeloid-lineage cells protects these cells from efficient HIV-1 infection, which likely prevents an innate immune response triggered by HIV-1. By contrast, SIVsm and HIV-2 encode Vpx to overcome SAMHD1-mediated restriction, which likely induces the innate antiviral immunity to confine viral infection in natural hosts. Thus, the interactions between SAMHD1 and Vpx may contribute to different consequences of HIV-1 and HIV-2 infection in humans (Table 1). Similarly, Manel and colleagues have suggested that HIV-1 restriction in DCs allows HIV-1 to avoid the antiviral immune responses derived from DCs, which are critical antigen presenting cells bridging the innate and adaptive immunity [12].

**Table 1:** Comparison of HIV-1 and HIV-2 regarding SAMHD1 degradation and potential disease consequences.

Lentiviruses	HIV-1	**SIVsm/HIV-2 **^**#**^
Vpx protein expression	No	Yes
Human SAMHD1 degradation	No	Yes
Efficient infection of myeloid cells	No	Yes
Triggering myeloid-cell-mediated innate anti-viral immunity through type I interferon	No	Yes
Potential disease outcome	Spread of infection and AIDS	Confined infection, no AIDS in natural hosts

^# ^It is known that cross-species transmission of sooty mangabey SIV (SIVsm) to humans has given rise to HIV-2 [6].

The biological function of SAMHD1 is largely unknown. *SAMHD1 *mutations are involved in Aicardi-Goutières syndrome (AGS), a genetic encephalopathy mimicking congenital viral infection [13]. *SAMHD1 *was initially cloned from human DCs as an interferon (IFN)-γ-inducible gene [14] and has been proposed to act as a negative regulator of the IFN response [13]. The cellular exonuclease TREX1 was recently shown to bind and digest excess cytosolic HIV-1 DNA that would otherwise activate type I IFN expression and trigger an innate immune response [15]. Interestingly, similar to *SAMHD1*, *TREX1 *mutations in humans are associated with autoimmune and inflammatory diseases, including AGS [15]. It is currently unknown whether polymorphisms of *SAMHD1 *and *TREX1 *are linked to AIDS progression or whether AGS patients are more susceptible to HIV-1 infection.

Three major retrovirus restriction factors have been identified: APOBEC3G, TRIM5α, and tetherin [6]. HIV-1 has developed mechanisms of evading these restriction factors mainly through its accessory proteins, such as Vif for APOBEC3G and Vpu for tetherin. These restriction factors function across many different cell types, whereas SAMHD1 appears to be specific to the myeloid-lineage cells. It might be possible that SAMHD1 acts in concert with another myeloid-specific co-factor [10]. It appears that only Vpx from the SIVsm/HIV-2 lineage counteracts SAMHD1-mediated HIV-1 restriction in myeloid cells [9,10], while HIV-1 Vpr does not interact or degrade SAMHD1 [10]. SAM domains are putative protein interaction modules that are capable of self-association and binding to RNA and non-SAM domain containing proteins. Given that SAMHD1 interferes with the accumulation of HIV-1 reverse transcripts, one can speculate that the SAM domain of SAMHD1 may bind HIV-1 RNA or proteins and mediate their degradation through the HD domain and the recruitment of the E3-ligase complex. Further delineation of the mechanisms of SAMHD1 restriction is required to fully understand the HIV-1 restriction in myeloid-lineage cells and why HIV-1 has not evolved a viral antagonist to counteract SAMHD1.

SAMHD1-mediated HIV-1 restriction has so far been analyzed only in monocyte-derived DCs [9] and macrophages [10], and it should be investigated in primary monocytes, myeloid DCs, as well as plasmacytoid DCs that can produce high levels of type I IFN upon HIV-1 stimulation [1,2]. It is unclear whether SAMHD1 is also type I IFN inducible, similar to other HIV-1 restriction factors. It would be interesting to know whether SAMHD1 can restrict other retroviruses, endogenous retroviruses, or other non-retroviruses and whether viruses use their own viral components to counteract SAMHD1.

## Conclusions

The discovery of SAMHD1 as a myeloid-cell-specific HIV-1 restriction factor opens many intriguing questions in understanding intrinsic immunity against HIV-1. When considering future therapeutic opportunities, enhancement of SAMHD1 function may help hosts develop potent innate and adaptive immune responses to HIV-1. Further investigation of the mechanisms underlying SAMHD1-mediated HIV-1 restriction will shed light on the innate immune response against retroviruses and aid in the future development of more effective anti-HIV-1 interventions.

## List of abbreviations

HIV-1: human immunodeficiency virus type 1; HIV-2: human immunodeficiency virus type 2; SIV: simian immunodeficiency virus; SAMHD1: sterile alpha motif domain- and HD domain-containing protein 1; DDB1: damage-specific DNA binding protein 1; CUL4A: Cullin-4A; DCAF1: DDB1- and CUL4A-associated factor-1; APOBEC3G: apolipoprotein B mRNA-editing, enzyme-catalytic, polypeptide-like 3G; TRIM5α: tripartite motif-containing protein 5α.

## Competing interests

The authors declare that they have no competing interests.

## Authors' contributions

Both authors contributed to the writing and editing of the manuscript and approved the final manuscript.
